# Supplementary material for: Empirical modeling of the percent depth dose for megavoltage photon beams
Source: PLoS One. 2022 Jan 6;17(1):e0261042. doi: 10.1371/journal.pone.0261042 (PMC8735605; doi:10.1371/journal.pone.0261042)
Supplement: Attachment — Submitted filename: point-to-point reply to reviewers comments 20211011.pdf [file pone.0261042.s002.pdf]

Reviewers' comments:

5. Review Comments to the Author

Reviewer #1:

This manuscript reports the development of an empirical method to model the high-energy photon beam percent depth dose (PDD) curve. It is an interesting and potentially beneficial for generating physical parameters used for monitor unit checking in radiation therapy. The accuracy of the mathematical formulation produced is very impressive.

Response to reviewer's comments: Thank you so much of your affirmation on this manuscript. The authors will do their best to correct every item suggested by the reviewer. Thank you again.

The manuscript in general is well written. The issue with the manuscript however, is with regard to the formatting and to a lesser extent, the language used. The authors should be more critical with the use of acronyms, and font formatting. For example:

• The acronym PDD should be used after it has been defined when it first appeared in the manuscript.

• Letter 'c' for Sc should be subscripted. 'Sc' should be used instead of 'collimator scatter' after it has been defined.

• Scalar quantity 'n' should be italised.

Response to reviewer's comments: Thank you for the suggestions. Any typo,

subscription after defined before appearance has been corrected and with yellow color highlighted in the context for reviewers easy to find. Thank you so much for your suggestions.

Some of the results are presented in the ‘Discussion’ section, which should go under the ‘Results’ section. More discussions on the methods and comparison with other methods/studies are desirable. The manuscript can also be benefited with proofreading exercise to polish up the language. Some other specific comments as follow:

Response to reviewer’s comments: Thank you for the suggestions. Some paragraphs will be re-examined and re-located to its proper position after all the corrections of the specific comments have been done and will be highlighted with yellow color in the context for reviewers easy to find. Thank you so much for your suggestions.

Abstract:

1. Page 3, line 54-55. Please acronymise percentage depth dose. Please check throughout the manuscript.

Response to reviewer’s comments: Thank you for the suggestions. All percentage depth dose has been acronymised to PDD when it has been defined after the first appearance and the corrections were also highlighted with yellow color throughout the manuscript for reviewers easy to find.

2. Page 3, line 57. Remove ‘collimator scatter’. Sc has been defined earlier. Please check throughout the manuscript.

Response to reviewer's comments: Thank you for the suggestions. All Collimator Scatter Factor has been acronymised to S_c when it has been defined after the first appearance and the corrections were also highlighted with yellow color throughout the manuscript for reviewers easy to find.

3. Page 4, line 73. Insert 'to' between 'sizes' and 'within'.

Response to reviewer's comments: Thank you for the suggestions. The "to" has been inserted between 'sizes' and 'within', the insertion was also highlighted with yellow color for reviewers easy to find. Thank you so much for the suggestion.

4. Page 4, line 77. Add 'setting' after 'clinical'.

Response to reviewer's comments: Thank you for the suggestions. The "setting" has been added after 'clinical', the correction was also highlighted with yellow color for reviewers easy to find. Thank you so much for the suggestion.

5. Page 4, line 77. Please subscript the letter 'c' in Sc. Check throughout manuscript.

Response to reviewer's comments: Thank you for the suggestions. The "c" has been subscripted in S_c, the correction was also highlighted with yellow color for reviewers easy to find. Thank you so much for the suggestion.

Introduction:

6. Page 6, line 100. Do not capitalise letter 'm' in measurement.

Response to reviewer's comments: Thank you for the correction. The "M" has been changed to "m", the correction was also highlighted with yellow color for reviewers easy to find. Thank you so much for the suggestion.

7. Page 6, line 103. Change 'on' to 'of'

Response to reviewer's comments: Thank you for the correction. The "on" has been changed to "of", the correction was also highlighted with yellow color for reviewers easy to find. Thank you so much for the suggestion.

8. Page 6 and 7, line 108-119. The authors presented the physics and a number of physical parameters of high energy x-ray which can be found in common radiation physics textbook, which I strongly feel is unnecessary. The authors should instead provide and explain the problem statement/study background or brief literature review of similar study, leading to the aims/objectives of the manuscript.

Response to reviewer's comments: Thank you for the suggestions.

The main purpose of this manuscript: The collection of clinical data for the implantation of treatment planning system for dose accuracy calculation is time consuming and repetitive. The achievement of this study provides a lot of help in double check of the measurement results to reduce the time of measurements, and the confidence to use interpolation. It would be helpful to know the smallest number of measurements needed to characterize the high-energy x-ray beams.

The study background or brief literature review of similar study in this manuscript: Several protocals provide a general framework and describes a large number of tests and procedures that should be considered by the users of RTPSs. However, the workload for the implementation of the recommendations from those documents are enormous and requires far more personnel and instrumentation resources than is available in most facilities, particularly within smaller hospitals. These hospitals are not always able to perform complete characterization, algorithm validation and software testing of dose calculation algorithms used in RTPS. These may include the S_c factors, also known as head scatter factors, which account for the variation in beam output with field size from changes in direct and indirect radiation from the head of the linear accelerator. The term S_{cp} contains both the collimator and phantom scatter that is defined as the ratio of dose for the field of interest to that of a reference field for the same delivered monitor units measured under full scatter conditions in a large water tank at the reference depth.

9. Page 7, line 127-133. The detailed explanation on the mathematical formula is not required here, as it is provided in the method. I suggest to just include the objective(s) of the current study, and its importance.

Response to reviewer's comments: Thank you for the suggestions. The original paragraph was corrected to be the following.

In this study, we proposed a simple mathematic equation to model the PDD by using the buildup-tail function. The quantity of interest is the parameters extracted from the well modeled PDD buildup-tail function for characterizing

the S_c either in a square field or in the different individual upper jaw and lower jaw setting separately for specific patient treatment monitor unit calculation.

The correction was also highlighted with yellow color for reviewers easy to find. Thank you so much for the suggestion.

Method:

10. Page 9, line 159. Change ‘Percent depth curve measurement’ to ‘Percent depth dose measurement’

Response to reviewer’s comments: Thank you for the suggestions.

The correction ”**II.A Percent depth dose measurement**” was also highlighted with yellow color for reviewers easy to find. Thank you so much for the suggestion.

11. Page 9, Line 163. Superscript number ‘3’ on cm3.

Response to reviewer’s comments: Thank you for the correction.

The original cm3 has been corrected to 0.125 cm^3, thank you for the correction.

12. Page 10, line 181 to 183. Please clarify the statement ‘This was performed by giving each film a priming dose of 2 Gy, to homogenize the film density using WHICH facility with a dose of 1 Gy at the photon energy of 6 MV’. I don’t understand what is mean by priming the film to 2 Gy, with a dose of 1 Gy?

Response to reviewer’s comments: Thank you for the suggestions. The pre-exposure technique on Gafchromic film we used was originated upon the article “An efficient protocol for radiochromic film dosimetry combining calibration and measurement in a single scan. Med Phys, 2012. [referenced at 10] ”. The original statement “This was performed by giving each film a priming dose of 2 Gy to homogenize the film density using WHICH facility with a dose of 1 Gy at the photon energy of 6 MV. We then measured the dose homogeneity using a densitometer.” was somehow redundant, therefore, it has been corrected to” This was performed by giving each film an initial dose of 2 Gy with a photon energy of 6 MV to homogenize the film polymer density in Gafchromic film experiment.

The correction was also highlighted with yellow color for reviewers easy to find. Thank you so much for the suggestion.

13. Page 10, line 190. Please specify the version and manufacturer of the software used.

Response to reviewer’s comments: Thank you for the suggestions. The version and manufacturer of the software has been descripted supplemently at the end of the original sentence as (PTW-Freiburg, Germany, VeriSoft version 3.1).

The correction was also highlighted with yellow color for reviewers easy to find. Thank you so much for the suggestion.

14. Page 10, line 191. Gafchromic film is specified as GAF film in some places, and back to Gafchromic at some places. Please be consistent.

The explanation of measurement using Gafchromic film in my opinion, is excessive. The method used for calibrating the film is widely published and the authors can just cite the reference(s), instead of explaining the procedures in great detail.

Response to reviewer's comments: Thank you for the suggestions.

The redundancy of the original sentence "Graded doses of 5, 10, 15, 40, 60, 80, 100, 150, and 200 cGy were given to the Gafchromic film to obtain the Hurter-Driffield calibration curve (H-D curve)[11]. All exposed films of depth dose curve were then scanned with an Epson Expression 11000XL scanner in the 48-bit RGB mode (16 bits per color), and the data were saved as tagged image file format (TIFF) and analyzed by the VeriSoft imaging procession software (PTW-Freiburg, Germany, VeriSoft version 3.1). A red filter was placed on top of the GAF films before scanning to increase the slope of the H-D curve, thereby raising the resolution of the dose-OD curves [12]" was streamlined to "The Hurter-Driffield calibration curve (H-D curve)[11] and the PDD films data scanned with a red filter [12] were analyzed by the VeriSoft imaging procession software (PTW-Freiburg, Germany, VeriSoft version 3.1) for further comparing with the depth dose curves measured by the

parallel-plate chamber.”

Results:

15. Page 14, line 246-247. It was stated that the measurement using ion chamber coincide with the film measurement. Please provide quantitative values on the agreement.

Response to reviewer’s comments: Thank you for the suggestions. The original sentence was added a supplementary quantitative value at the end as” The dose variations of PDD of high energy photon beams measured by films and by ion chamber were less than 0.5%.”

The correction was also highlighted with yellow color for reviewers easy to find. Thank you so much for the suggestion.

16. Page 14, line 249. Change ‘at the commissioning’ to ‘during commissioning’.

Response to reviewer’s comments: Thank you for the suggestions. The correction has been made and highlighted with yellow color for reviewers easy to find. Thank you so much for the suggestion.

17. Page 15-16, Figure 1(a)-(d). Despite the excellent agreement between the fitting and measurement, it is imperative to provide the values of goodness of fit (or other quantitate deviation measurement) as a quantitative measure on the fitting performance.

Response to reviewer’s comments: Thank you for the suggestions. Besides the original description in the result section, the additional table has been inserted to support the quantitative performance of the modeling in describing the fitting of PDD.

Please check the comparison tabulated at the following table, thank you so much for the suggestion to make the manuscript more readable.

Table 1. The comparison of four high-energy photon measured and modeled PDD with two published and two facility data

	4 MV photon (published data)			6 MV photon (facility data)			10 MV photon (facility data)			18 MV photon (published data)		
depth (cm)	measured PDD	modeled PDD	error (%)	measured PDD	modeled PDD	error (%)	measured PDD	modeled PDD	error (%)	measured PDD	modeled PDD	error (%)
0.1	38.89	29.46	-24.24	40.12	23.28	-41.97	38.98	17.00	-56.39	32.20	10.94	-66.03
1	98.67	98.75	0.09	98.09	97.12	-0.99	93.91	90.70	-3.42	76.55	78.09	2.01
1.5	98.80	98.92	0.12	100.08	100.08	0.00	97.39	95.82	-1.62	89.55	91.59	2.27
2	98.44	98.59	0.15	99.62	99.56	-0.07	100.02	99.80	-0.22	96.07	97.39	1.38
2.5	96.17	96.32	0.16	98.08	97.82	-0.27	99.22	99.24	0.02	99.01	99.59	0.58
3	93.78	93.93	0.16	96.10	95.66	-0.45	97.96	98.23	0.27	100.00	100.00	0.00
3.5	91.21	91.36	0.16	93.89	93.33	-0.59	96.01	96.52	0.53	99.33	99.48	0.15
4	88.65	88.80	0.17	91.59	90.95	-0.70	94.04	94.63	0.63	98.42	98.43	0.01
4.5	86.10	86.25	0.17	89.25	88.56	-0.78	92.04	92.62	0.63	97.21	97.09	-0.12
5	83.61	83.76	0.18	86.92	86.19	-0.84	90.01	90.57	0.61	95.40	95.57	0.18
5.5	81.14	81.29	0.18	84.62	83.86	-0.89	87.98	88.50	0.59	93.58	93.95	0.39
6	78.78	78.93	0.19	82.35	81.58	-0.93	85.97	86.44	0.55	92.09	92.27	0.20
6.5	76.44	76.59	0.20	80.12	79.35	-0.97	83.87	84.40	0.64	90.15	90.56	0.46
7	74.19	74.34	0.20	77.94	77.17	-0.99	81.38	82.40	1.25	88.34	88.84	0.57
7.5	71.99	72.14	0.21	75.82	75.04	-1.02	79.14	80.43	1.63	86.57	87.12	0.64
8	69.85	70.00	0.21	73.74	72.97	-1.04	77.27	78.50	1.58	84.72	85.41	0.82
8.5	67.78	67.93	0.22	71.71	70.96	-1.05	75.44	76.60	1.54	83.05	83.71	0.80
9	65.75	65.90	0.23	69.74	68.99	-1.07	73.64	74.74	1.50	81.31	82.04	0.89
9.5	63.81	63.96	0.24	67.81	67.08	-1.08	71.89	72.93	1.45	79.68	80.39	0.89
10	61.89	62.04	0.24	65.94	65.22	-1.09	70.16	71.14	1.40	78.07	78.76	0.88
10.5	60.06	60.21	0.25	64.12	63.41	-1.10	68.50	69.43	1.35	76.52	77.16	0.83
11	58.26	58.41	0.26	62.34	61.65	-1.10	66.85	67.72	1.31	74.83	75.58	1.01
11.5	56.54	56.69	0.27	60.61	59.94	-1.11	65.26	66.08	1.26	73.31	74.03	0.99
12	54.84	54.99	0.27	58.93	58.27	-1.12	63.69	64.46	1.21	71.74	72.52	1.07
12.5	53.20	53.35	0.28	57.30	56.66	-1.12	62.17	62.89	1.16	70.28	71.04	1.08
13	51.58	51.73	0.29	55.71	55.08	-1.13	60.68	61.35	1.11	68.81	69.56	1.09
13.5	49.96	50.11	0.30	54.16	53.55	-1.13	59.21	59.84	1.06	67.40	68.14	1.10
14	48.35	48.50	0.31	52.66	52.06	-1.14	57.80	58.38	1.01	65.99	66.72	1.11
14.5	46.73	46.88	0.32	51.20	50.61	-1.14	56.39	56.93	0.96	64.65	65.35	1.09
15	45.11	45.26	0.33	49.77	49.20	-1.14	55.05	55.55	0.91	63.30	63.99	1.08
15.5	43.50	43.65	0.34	48.39	47.84	-1.15	53.72	54.18	0.86	61.99	62.68	1.10
16	41.88	42.03	0.36	47.04	46.50	-1.15	52.44	52.86	0.81	60.68	61.36	1.13

16.5	40.26	40.41	0.37	45.74	45.21	-1.15	51.17	51.56	0.76	59.43	60.10	1.14
17	38.65	38.80	0.39	44.46	43.95	-1.15	49.94	50.30	0.71	58.18	58.84	1.15
17.5	37.03	37.18	0.41	43.23	42.73	-1.16	48.74	49.07	0.66	56.98	57.63	1.15
18	35.42	35.57	0.42	42.03	41.54	-1.16	47.56	47.86	0.61	55.78	56.42	1.15
18.5	33.80	33.95	0.44	40.86	40.38	-1.16	46.43	46.69	0.56	54.61	55.26	1.19
19	32.18	32.33	0.47	39.72	39.26	-1.16	45.30	45.53	0.51	53.45	54.10	1.23
19.5	30.57	30.72	0.49	38.62	38.17	-1.16	44.22	44.42	0.46	52.31	52.99	1.30
20	28.95	29.10	0.52	37.54	37.10	-1.16	43.15	43.32	0.41	51.17	51.88	1.37
20.5	27.34	27.49	0.55	36.50	36.07	-1.16	42.11	42.27	0.36	50.09	50.81	1.42
21	25.72	25.87	0.58	35.48	35.07	-1.17	41.10	41.23	0.31	49.02	49.74	1.47
21.5	24.10	24.25	0.62	34.49	34.09	-1.17	40.11	40.21	0.26	47.99	48.71	1.50
22	22.49	22.64	0.67	33.53	33.14	-1.17	39.14	39.23	0.21	46.97	47.69	1.53
22.5	20.87	21.02	0.72	32.60	32.22	-1.17	38.20	38.26	0.16	46.01	46.71	1.52
23	19.25	19.40	0.78	31.69	31.32	-1.17	37.28	37.32	0.11	45.05	45.72	1.50

18. Page 17, line 305-309. The expression of Sc should be provided in the methods section. Is ‘FS’ the dimension of field size? This should be stated.

Response to reviewer’s comments: Thank you for the suggestions. Please allow me to rebuttal the reviewer’s suggestion that equation 2, $S_{c,E} = n_E \cdot (FS)^{0.63\mu_E}$, which was expressed by using the parameters n and μ after the best fitting of the empirical buildup-tail function, since there were no fitting result in method section, therefore, please accept authors opinions to leave equation 2 at III B in Result section. Thank you for your understanding.

The explanation of FS has been added to the end of the sentence.

$S_{c,E} = n_E \cdot (FS)^{0.63\mu_E}$ ………………………………. equation 2

n_E and μ_E denote the parameters n and μ in empirical buildup-tail function at

photon energy E, while FS stand for field size of interest.

19. Page 18, caption for Figure 2. It was stated that ‘the Sc of photon energy 6 MV (a) and 10 MV 321 (b) can be expressed perfectly by using the parameters n and μ’. I disagree to that because albeit from a reasonably good fit, it is not perfect. There are some slight disagreements at certain field sizes.

Response to reviewer’s comments: Thank you for the comment. Indeed, the author thanks the reviewer’s correction. The caption of Figure 2 was corrected to be the following. Thank you so much for the opinion.

Fig. 2 The S_c of photon energy 6 MV (a) and 10 MV (b) can be expressed in a certain acceptable deviation within 0.8% by using the parameters n and μ modeled as a function of field size in empirical Buildup-tail function by the equation of $S_{c,E} = n_E \cdot (FS)^{0.63\mu_E}$, n_E and μ_E denote the parameters n and μ in empirical Buildup-tail function at photon energy E, FS denotes the field size.

20. Page 19, Table 2. I noted the deviations for 6 MV are in general larger than 10 MV. Any explanations for this?

Response to reviewer’s comments: Thank you for the question. To the author’s best knowledge, the concept of in-air output ratio S_c was introduced to

characterize how the incident photon fluence per monitor unit varies with collimator settings, and the author believe the S_c of a larger photon energy is more sensitive in accordance with the field size, namely, the larger collimator setting, the more S_c it is. This might be owing to the strong side scatter of the higher energy of photons. As we can see in Fig. 2b, the S_c of 10 MV increases gradually when the field sizes open widely, on the contrary, Fig. 2a, the S_c of 6 MV becomes slightly saturation when the field sizes open widely. This phenomenon causes the curvature of S_c of photon energy 10 MV to be easy fitted by the parameters n and μ as a function of field size than in a photon energy of 6 MV. Hope this explanation can be recognized by the reviewer. Thank you so much for the question.

Discussion:

Some of the results presented here should go under the 'Results' section. For instance, page 21, lines

21. Page 21, lines 383-390.

Response to reviewer's comments: These statements have been moved to III B under Results section and highlighted with yellow color for reviewers easy to find. Thank you so much for the suggestion.

22. Page 24, line 440. Change 'interest' to 'interesting'.

Response to reviewer's comments: The correction has been made and highlighted with yellow color for reviewers easy to find. Thank you so much for the suggestion.

23. Page 24, line 440-442. It was stated that 'It is very interest that a weaker variation can be seen when the upper jaw and lower jaw collimator setting was reversed'. Any plausible reason/explanation for this?

Response to reviewer's comments:

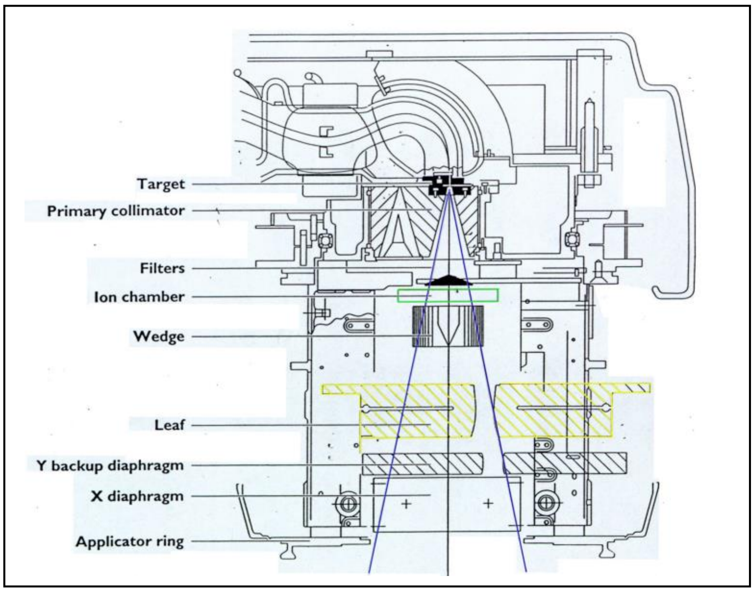

According to the Elekta head structure shown above, we can see the upper jaw are composed by the leaf and the Y backup diaphragm, while the low jaw is only a X diaphragm. As the example demonstrated in manuscript, the S_c of

upper jaw x lower jaw setting at 10 cm x 15 cm and 15 cm x 10 cm was 1.0049, 1.0104 in Table 4. Here, it is explicitly a wide upper jaw of 15 cm of the upper jaw by lower jaw setting at 15 cm x 10 cm can provide more scatter photon than an upper jaw setting of 10 cm in 10 cm x 15 cm does. No wonder the S_c of upper jaw at 15 cm x 10 cm have a larger value of 1.0104 than that of 1.0049 provided by 10 cm x 15 cm.

24. Some tables here (Figure 3 and 4) are results and should go under the results section.

Response to reviewer's comments: Please allow me to clarify there are only three figures. Figure 1 describes the best fitting of photon PDD curve at energy 4, 6, 10 and 18 MV. Figure 2 describes the S_c of photon energy 6 and (2a) 10 MV(2b) can be expressed by using the parameters n and μ modeled in empirical buildup-tail function by the equation of $S_{c,E} = n_E \cdot (FS)^{0.63\mu_E}$. Figure 1 and 2 are in Results section.

As for Figure 3, this figure discusses the function of parameter n in the photon beam of a hardening factor in the buildup, the larger n (n= 4.95) the high beam quality, thus accompany the less surface dose and the deeper d_{max} (to compare n = 4.95 and n=0.0495 in Fig. 2). The parameter μ represents

the attenuation coefficient of the photon beam in a medium. The larger μ (μ = 0.458) the more attenuation, thus, the steeper curves and to have a shortened range (to compare μ= 0.458 and μ= 0.00458).

Therefore, the author asks the reviewer's opinion to leave the Figure 3 in Disscussion section to its proper position, thank you so much.

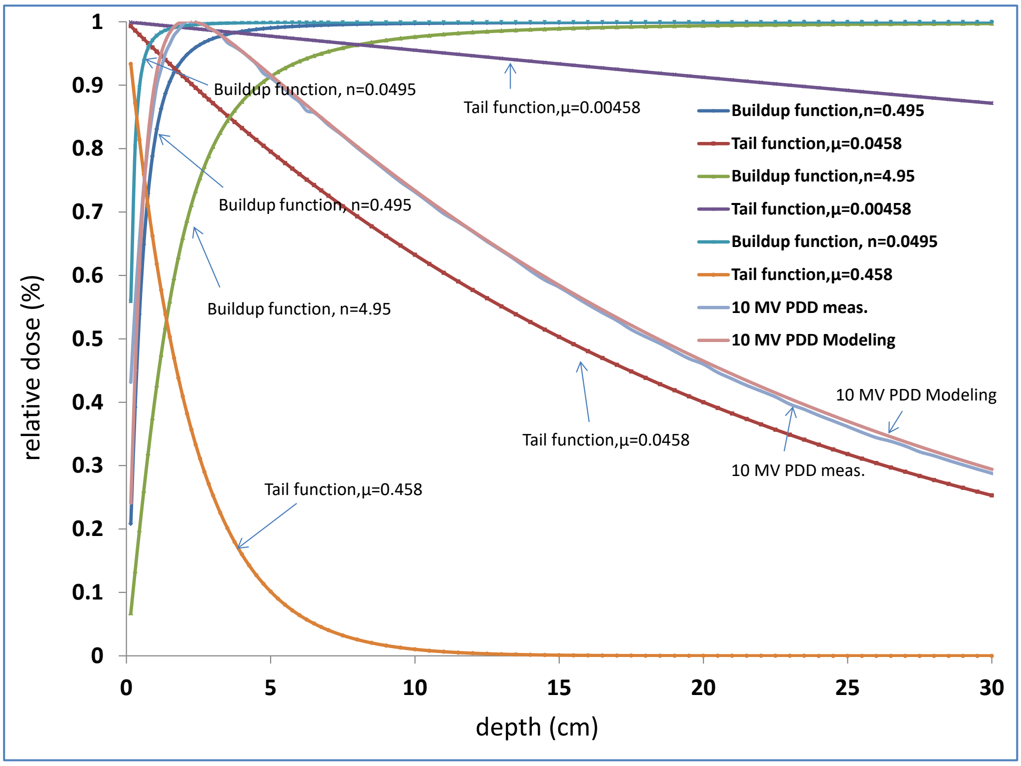

Fig. 3

25. More discussion on the utility of the empirical mathematical formula is desirable.

Are there any similar empirical methods/mathematical formula reported in the literature?

Response to reviewer’s comments: So far as I can reach, the most representative report is the author Timothy C. Zhu’s paper entitled “A three-parameter semiempirical model for scatter-to-primary dose ratio SPR to fit PDD. There were also a very complicate numerical method to use Monte Carlo(MC) by calculating SPR for photon energy spectrum to model the PDD of Co-60and 24 MV.

Some of the articles model the photon PDD by using four parameters equation empirically as the form below, and the fitting parameters are listed below,

S_c or $S_{cp}=aS^{-0.5}+b/S+c\ \mathrm{ln}S^2+d$

Table of the measured quality indices and 10-cm deep PDD values (100-cmSSD, collimator setting of 10 x 10 cm^2) for the flattened beams studied in their work. The coefficients a, b, c, an d for collimator and total scatter data fits to the measured data using equation above, are given for the 3 beams investigated

	6 MV Varian	6 MV Elekta	10 MV Varian
$TPR_{20/10}$	0.664	0.684	0.736
PDD_{10}	66.50%	67.80%	73.50%
S_c fit parameters			
a	0.8384	0.1178	1.4273
b	0.5389	0.0259	0.9098
c	0.0194	0.0022	0.0425
d	1.3004	1.0448	1.556

We conclude our method proposed in this study is relatively simple and

pratical in clinical use.

26. What are the benefits of this method for generating physical parameters for MU checking?

Response to reviewer's comments:

The most benefit of S_c modeled in this study can be used in dose calculation as described in TG74. It is suitable for TPR-based MU calculation algorithm where the reference depth is typically 10 cm or beyond electron contamination. The "collimator-scatter factor" measured at d_{max} is used for TMR-based MU calculation algorithm. In this use, TG74 recommend using S_c in-air output ratio as defined in the report so long as S_p=S_{cp} /S_c is determined using S_{cp} measured at d_{max}.

ACKNOWLEDGMENTS

27. What is the future work/recommendations as I believe more work is required to verified the accuracy/feasibility of using such formula in clinical setting?

Response to reviewer's comments:

As we all know, to collect those data for the implantation of treatment planning

system for dose accuracy calculation is time consuming and repetitive. In order to plan the measurements, to use interpolation, and to double check the measured results, the method provided in this study would be helpful to know the smallest number of measurements needed to characterize the high-energy x-ray beams

The future work is to parameterize our flattened filter free beams using the simple mathematical expression proposed in this study. The data reproduced may be used as expectation values for comparison with commissioning beams,

As for the feasibility of using such formula in clinical setting, once the parameters n and μ was generated by the empirical function from the best fitting of high energy photon beams, the S_c can then be expressed in a certain acceptable deviation within 0.8% by the parameters n and μ as a function of field size in empirical Buildup-tail function by equation:

$S_{c,E} = n_E \cdot (FS)^{0.63\mu_E}$, then all the calculated S_c are used to provide a solution for double checking the measured S_c.

The author would like to express many thanks of the reviewer's time and assistance on the manuscript of the improvement for better understand.

Reviewer #2: Title: Empirical Modeling of the Percent Depth Dose for Megavoltage Photon Beams

Manuscript number: PONE-D-21-29815

General comments:

This paper describes an empirical method to model the PDD of the high-energy photon beam using a mathematical – buildup-tail function. The study was interesting because the proposed mathematical model could be useful to predict the collimator scatter factor, Sc for PDDs.

Please clarify if the measured PDD in Fig 1a-d, were PDD from published literature or from ion chamber measurements. If they were from published data, how did the author managed to acquire such detail of individual measurement points?

Response to reviewer's comments: Thank you for the question.

Since we have only two high-energy photon beams of 6 and 10 MV, in order to test the power of our home-generated empirical function, we adopted two published photon energy's PDD data . The author have described the details in **Experiment Design and Steps** section that the PDD for four high-energy photon energies of 4 MV, 6 MV, 10 MV, 18 MV were modeled in this study. The measurements data were 6 MV, 10 MV while 4 MV and 18 MV were published data [7, 8].

The detail individual measurement points of two published

high-energy photon beams were copy from the published tables.

There are numerous language and grammatical errors throughout the manuscript, unnecessary capitalization at random places, subscripts for Sc etc should be carefully pick-up and corrected by the authors.

Response to reviewer's comments: Thank you for the comments. All of the shortcoming will be corrected and the final revised manuscript will be edited by a professional English proof reading then re-submit.

Specific comments:

Introduction –

1. Could the authors present the background and the need for this study? Because, typically PDDs and Sc are measured from the actual beam, because the beams are different for every linac, and depending on the detector used to measure it.

Response to reviewer's comments: Thank you for the comments.

The main purpose of this manuscript: The collection of clinical data for the implantation of treatment planning system for dose accuracy calculation is time

consuming and repetitive. The achievement of this study provides a lot of help in double check of the measurement results to reduce the time of measurements, and the confidence to use interpolation. It would be helpful to know the smallest number of measurements needed to characterize the high-energy x-ray beams.

2. Line 100 – measurement – do not capitalize “M”

Response to reviewer’s comments: Thank you for the correction. The “M” has been changed to “m”, the correction was also highlighted with yellow color for reviewers easy to find. Thank you so much for the suggestion.

3. Line 149 and many other places – Sc - to make sure the c is subscripted

Response to reviewer’s comments: Thank you for the suggestions. Any typo, subscription after defined before appearance has been corrected and with yellow color highlighted in the contexts for reviewers easy to find. Thank you so much for your suggestions.

4. Line 153 – four photon energies

Response to reviewer’s comments: Thank you for the corrections. The correction has been made to “energies” and “was” was corrected to “were”,

and with green color highlighted in the contexts for reviewers easy to find. Thank you so much for your suggestions.

5. Line 159 – Percent depth dose curve

Response to reviewer's comments: Thank you for the suggestions.

The correction "**II.A Percent depth dose measurement**" was also highlighted with yellow color for reviewers easy to find. Thank you so much for the suggestion.

6. Line 163 – please specify the company, state and country of the PTW. The volume is cm3.

Response to reviewer's comments: Thank you for the suggestions.

The correction was also highlighted with green color for reviewers easy to find. Thank you so much for the suggestion.

(PTW ionization Freiburg Gmbh, type 31010, volume 0.125 cm^3).

7. Line 182 – What is the reason to prime the film for 2Gy? Was it 1Gy or 2Gy? Please clarify? If the films are primed with 1-2 Gy, then, how would that affect the calibration curves? Where is the 0 Gy point?

Response to reviewer’s comments: Thank you for the suggestions.

The pre-exposure technique on Gafchromic film we used was originated upon the article “An efficient protocol for radiochromic film dosimetry combining calibration and measurement in a single scan. Med Phys, 2012. [referenced at 10] ”. The original statement “This was performed by giving each film a priming dose of 2 Gy to homogenize the film density using WHICH facility with a dose of 1 Gy at the photon energy of 6 MV. We then measured the dose homogeneity using a densitometer.” was somehow redundant, therefore, it has been corrected to” This was performed by giving each film an initial dose of 2 Gy with a photon energy of 6 MV to homogenize the film polymer density in Gafchromic film experiment.

Where is the 0 Gy point? The pre-exposure technique is to smooth the polymer inhomogeneity density of the film. The zero Gy point is the point to remove the optic density of pre-exposed 2 Gy dose.

The correction was also highlighted with yellow color for reviewers easy to find. Thank you so much for the suggestion.

8. Line 186 – Why is the gafchromic calibration curve called a Hurter-Diffield calibration curve?

Response to reviewer’s comments: Thank you for the question.

The full name of well-known H-D curve (film optical density calibration curve) is Hurter-Diffield calibration curve.

9. There were description of the use of gaf film – but I fail to see the purpose of the film measurements, since the authors only used the ion chamber measured data.

Response to reviewer's comments: Thank you for the questions.

Since the use of film dosimetry is quite common, the comparison of Gafchromic and ion chamber in PDD measurements were presented by text description to be more concise in III A Results section.

10. Line 200 – 214 – Please explain the linac used to measure the Sc.

Response to reviewer's comments: Thank you for the questions. The Linacs have been inserted to the text as the following,

The measurement of S_c for Elekta infinity and Varian trueBeam with the high-energy photon energy of 6 and 10 MV were conducted using a PTW Semiflex chamber.

Although two Linac Accelerators were conducted in S_c

measurements, the major analysis of S_c focus yet on Elekta in this study due to the future study will be on the characteristic study of S_c in FF and FFF of Varian trueBeam.

11. Line 222 – no need to capitalize percent depth dose

Response to reviewer's comments: Thank you for the questions.

The Percent Depth Dose has been abbreviated to PDD, the correction was also highlighted with yellow color for reviewers easy to find. Thank you so much for the suggestion.

12. Line 237 – four photon energies

Response to reviewer's comments: Thank you for the questions.

The correction was made and also highlighted with green color for reviewers easy to find. Thank you so much for the correction.

13. Line 240 – please write in full sentence.

Response to reviewer's comments: Thank you for the questions.

The title of **III.A.** has been corrected to a full sentence, and the correction was also highlighted with green color for reviewers easy to

find. Thank you so much for the correction.

14. Figure 1a – d – The two modeled and published PDD appears to match very well, but, at the region of dose buildup and end of tail, the dose descrepencies are obvious. Perhaps an additional graph on the error/difference between the 2 curves can be added to the figures to show the differences?

Response to reviewer’s comments: Thank you for the questions.

Please check the comparison tabulated at the following table, this table was also entitled to Table 1 in the context. Thank you so much for the suggestion to make the manuscript more readable.

Table 1. The comparison of four high-energy photon measured and modeled PDD with two published and two facility data

	4 MV photon (published data)			6 MV photon (facility data)			10 MV photon (facility data)			18 MV photon (published data)		
depth (cm)	measured PDD	modeled PDD	error (%)	measured PDD	modeled PDD	error (%)	measured PDD	modeled PDD	error (%)	measured PDD	modeled PDD	error (%)
0.1	38.89	29.46	-24.24	40.12	23.28	-41.97	38.98	17.00	-56.39	32.20	10.94	-66.03
1	98.67	98.75	0.09	98.09	97.12	-0.99	93.91	90.70	-3.42	76.55	78.09	2.01
1.5	98.80	98.92	0.12	100.08	100.08	0.00	97.39	95.82	-1.62	89.55	91.59	2.27
2	98.44	98.59	0.15	99.62	99.56	-0.07	100.02	99.80	-0.22	96.07	97.39	1.38
2.5	96.17	96.32	0.16	98.08	97.82	-0.27	99.22	99.24	0.02	99.01	99.59	0.58
3	93.78	93.93	0.16	96.10	95.66	-0.45	97.96	98.23	0.27	100.00	100.00	0.00
3.5	91.21	91.36	0.16	93.89	93.33	-0.59	96.01	96.52	0.53	99.33	99.48	0.15
4	88.65	88.80	0.17	91.59	90.95	-0.70	94.04	94.63	0.63	98.42	98.43	0.01
4.5	86.10	86.25	0.17	89.25	88.56	-0.78	92.04	92.62	0.63	97.21	97.09	-0.12
5	83.61	83.76	0.18	86.92	86.19	-0.84	90.01	90.57	0.61	95.40	95.57	0.18
5.5	81.14	81.29	0.18	84.62	83.86	-0.89	87.98	88.50	0.59	93.58	93.95	0.39
6	78.78	78.93	0.19	82.35	81.58	-0.93	85.97	86.44	0.55	92.09	92.27	0.20
6.5	76.44	76.59	0.20	80.12	79.35	-0.97	83.87	84.40	0.64	90.15	90.56	0.46
7	74.19	74.34	0.20	77.94	77.17	-0.99	81.38	82.40	1.25	88.34	88.84	0.57
7.5	71.99	72.14	0.21	75.82	75.04	-1.02	79.14	80.43	1.63	86.57	87.12	0.64
8	69.85	70.00	0.21	73.74	72.97	-1.04	77.27	78.50	1.58	84.72	85.41	0.82
8.5	67.78	67.93	0.22	71.71	70.96	-1.05	75.44	76.60	1.54	83.05	83.71	0.80
9	65.75	65.90	0.23	69.74	68.99	-1.07	73.64	74.74	1.50	81.31	82.04	0.89
9.5	63.81	63.96	0.24	67.81	67.08	-1.08	71.89	72.93	1.45	79.68	80.39	0.89
10	61.89	62.04	0.24	65.94	65.22	-1.09	70.16	71.14	1.40	78.07	78.76	0.88
10.5	60.06	60.21	0.25	64.12	63.41	-1.10	68.50	69.43	1.35	76.52	77.16	0.83
11	58.26	58.41	0.26	62.34	61.65	-1.10	66.85	67.72	1.31	74.83	75.58	1.01
11.5	56.54	56.69	0.27	60.61	59.94	-1.11	65.26	66.08	1.26	73.31	74.03	0.99
12	54.84	54.99	0.27	58.93	58.27	-1.12	63.69	64.46	1.21	71.74	72.52	1.07
12.5	53.20	53.35	0.28	57.30	56.66	-1.12	62.17	62.89	1.16	70.28	71.04	1.08
13	51.58	51.73	0.29	55.71	55.08	-1.13	60.68	61.35	1.11	68.81	69.56	1.09
13.5	49.96	50.11	0.30	54.16	53.55	-1.13	59.21	59.84	1.06	67.40	68.14	1.10
14	48.35	48.50	0.31	52.66	52.06	-1.14	57.80	58.38	1.01	65.99	66.72	1.11
14.5	46.73	46.88	0.32	51.20	50.61	-1.14	56.39	56.93	0.96	64.65	65.35	1.09
15	45.11	45.26	0.33	49.77	49.20	-1.14	55.05	55.55	0.91	63.30	63.99	1.08
15.5	43.50	43.65	0.34	48.39	47.84	-1.15	53.72	54.18	0.86	61.99	62.68	1.10
16	41.88	42.03	0.36	47.04	46.50	-1.15	52.44	52.86	0.81	60.68	61.36	1.13

16.5	40.26	40.41	0.37	45.74	45.21	-1.15	51.17	51.56	0.76	59.43	60.10	1.14
17	38.65	38.80	0.39	44.46	43.95	-1.15	49.94	50.30	0.71	58.18	58.84	1.15
17.5	37.03	37.18	0.41	43.23	42.73	-1.16	48.74	49.07	0.66	56.98	57.63	1.15
18	35.42	35.57	0.42	42.03	41.54	-1.16	47.56	47.86	0.61	55.78	56.42	1.15
18.5	33.80	33.95	0.44	40.86	40.38	-1.16	46.43	46.69	0.56	54.61	55.26	1.19
19	32.18	32.33	0.47	39.72	39.26	-1.16	45.30	45.53	0.51	53.45	54.10	1.23
19.5	30.57	30.72	0.49	38.62	38.17	-1.16	44.22	44.42	0.46	52.31	52.99	1.30
20	28.95	29.10	0.52	37.54	37.10	-1.16	43.15	43.32	0.41	51.17	51.88	1.37
20.5	27.34	27.49	0.55	36.50	36.07	-1.16	42.11	42.27	0.36	50.09	50.81	1.42
21	25.72	25.87	0.58	35.48	35.07	-1.17	41.10	41.23	0.31	49.02	49.74	1.47
21.5	24.10	24.25	0.62	34.49	34.09	-1.17	40.11	40.21	0.26	47.99	48.71	1.50
22	22.49	22.64	0.67	33.53	33.14	-1.17	39.14	39.23	0.21	46.97	47.69	1.53
22.5	20.87	21.02	0.72	32.60	32.22	-1.17	38.20	38.26	0.16	46.01	46.71	1.52
23	19.25	19.40	0.78	31.69	31.32	-1.17	37.28	37.32	0.11	45.05	45.72	1.50

15. Figure 2 a and b – again, what are the errors of the measured values, i.e. error bars? Where were these values obtained from?

Response to reviewer's comments: Thank you for the questions.

The errors of the measured values were within ± 0.5%, and the Fig.2a and 2b show the best fitting of the S_c by the $S_{c,E}$ equation.

$S_{c,E} = n_E \cdot (FS)^{0.63\mu_E}$ …………………………….. equation 2

n_E and μ_E denote the parameters n and μ in empirical buildup-tail function at photon energy E, while FS stand for field size of interest.

The S_c of photon energy 6 and 10 MV can be expressed in a certain acceptable deviation within 0.8% by the parameters n and μ as a function of field size in empirical buildup-tail function by the equation:

16. Table 1 – These parameters are specific for the 4 PDDs that the authors had modeled on. Please discuss how these values are going to be applicable for any PDDs?

Response to reviewer's comments: Thank you for the questions. Firstly, all new linear accelerator's PDD data have to be fitted by using the home-generated buildup-tail function to get the optimal parameters n and μ. Secondly, to input the optimal parameters n and μ into the equation, $S_{c,E} = n_E \cdot (FS)^{0.63\mu_E}$, for calculating the S_c of any photon energy.

Please allow the author to emphasize one point, the measurement of Linac clinical data is time consuming, repetitive and often with an errancy. The achievement of this study here can help the user to do the double check of the clinical measurements, it would be helpful to know the smallest number of measurements needed to characterize the high-energy photon beams.

17. Line 306 – no need to capitalize buildup

Response to reviewer's comments: Thank you for the questions. The "Buildup-tail function" has been corrected to "buildup-tail

function, and the correction was also highlighted with green color for reviewers easy to find. Thank you so much for the correction.

18. Table 2 – shows the error between the measured and predicted Sc. Perhaps, displaying this in a graph format would be better and summarize the average, SD or max deviation at what field size. Unless the numerical value has any value for any PDD or field sizes of the reader.

Response to reviewer's comments: Thank you for the questions.

Thank you for the suggestion. The original Table 2 has been re-arranged to Table 3 owing to a new Table was inserted according to the other reviewer's suggestion, and all data have been inserted SD which were calculated from the raw data.

Thank you for the suggestion again.

19. Line 359 – no need to capitalize buildup

Response to reviewer's comments: Thank you for the questions.

Again, the "Buildup-tail function" has been corrected to "buildup-tail function, and the correction was also highlighted with green color for

reviewers easy to find. Thank you so much for the correction.

20. Interesting explanation of the equations. However, one of the limitation of the model, correct me if I am wrong, is unable to predict the dose at the surface, at d = 0 cm and the discrepancy of the model at the dose buildup region could be quite big. Can the authors comment on the errors at these regions?

Response to reviewer’s comments: Thank you for the questions.

Indeed, the reviewer’s comments on these points are correct. The prediction of dose at the surface as well as the buildup region is not satisfied in buildup-tail function. However, the author are trying to modified the function with some scatter coefficient to control the dose near superficial region to improve the limitation of this function. Thank you so much of your valuable question.

21. Line 427 – no figure 3 caption.

Response to reviewer’s comments: Thank you for the questions.

I think the original caption of figure 3 might be hidden in the reviewer’s version. Please check if any problem on the caption of

figure 3 of this revised version, thank you so much of the question.

Fig. 3

The parameter n represents the photon beam hardening factor in the buildup, the larger n (n= 4.95) the high beam quality, thus accompany the less surface dose and the deeper d_{max} (to compare n = 4.95 and n=0.0495 in Fig. 2). The parameter μ represents the attenuation coefficient of the photon beam in the medium. The larger μ (μ = 0.458) the more attenuation, thus, the steeper curves and to have a shortened range (to compare μ= 0.458 and μ= 0.00458).

22. Table 3 – should this be in the results?

Response to reviewer's comments: Thank you for the questions.

Thank you for the suggestion, the author will move this table to the place where it belongs.

The authors would like to express their appreciation for the reviewer's time and assistance on the manuscript. We sincerely hope this manuscript would be recognized by the reviewers and have the honor to be published on your distinguish journal.
